# ALKBH8 contributes to neurological function through oxidative stress regulation

**DOI:** 10.1093/pnasnexus/pgae115

**Published:** 2024-03-28

**Authors:** Kohei Honda, Hiroaki Hase, Sayaka Tanikawa, Katsuya Okawa, Lu Chen, Takumi Yamaguchi, Manami Nakai, Kaori Kitae, Yukio Ago, Shinsaku Nakagawa, Kazutake Tsujikawa

**Affiliations:** Laboratory of Molecular and Cellular Physiology, Graduate School of Pharmaceutical Sciences, Osaka University, Suita, Osaka 565-0871, Japan; Laboratory of Molecular and Cellular Physiology, Graduate School of Pharmaceutical Sciences, Osaka University, Suita, Osaka 565-0871, Japan; Laboratory of Molecular and Cellular Physiology, Graduate School of Pharmaceutical Sciences, Osaka University, Suita, Osaka 565-0871, Japan; Laboratory of Molecular and Cellular Physiology, Graduate School of Pharmaceutical Sciences, Osaka University, Suita, Osaka 565-0871, Japan; Laboratory of Biopharmaceutics, Graduate School of Pharmaceutical Sciences, Osaka University, Suita, Osaka 565-0871, Japan; Laboratory of Biopharmaceutics, Graduate School of Pharmaceutical Sciences, Osaka University, Suita, Osaka 565-0871, Japan; Laboratory of Molecular and Cellular Physiology, Graduate School of Pharmaceutical Sciences, Osaka University, Suita, Osaka 565-0871, Japan; Laboratory of Molecular and Cellular Physiology, Graduate School of Pharmaceutical Sciences, Osaka University, Suita, Osaka 565-0871, Japan; Laboratory of Biopharmaceutics, Graduate School of Pharmaceutical Sciences, Osaka University, Suita, Osaka 565-0871, Japan; Global Center for Medical Engineering and Informatics, Osaka University, Suita, Osaka 565-0871, Japan; Department of Cellular and Molecular Pharmacology, Graduate School of Biomedical and Health Sciences, Hiroshima University, Hiroshima 734-8553, Japan; Laboratory of Biopharmaceutics, Graduate School of Pharmaceutical Sciences, Osaka University, Suita, Osaka 565-0871, Japan; Global Center for Medical Engineering and Informatics, Osaka University, Suita, Osaka 565-0871, Japan; Laboratory of Molecular and Cellular Physiology, Graduate School of Pharmaceutical Sciences, Osaka University, Suita, Osaka 565-0871, Japan

## Abstract

Transfer RNA (tRNA) modification is essential for proper protein translation, as these modifications play important roles in several biological functions and disease pathophysiologies. AlkB homolog 8 (ALKBH8) is one of the nine mammalian ALKBH family molecules known to regulate selenoprotein translation through the modification of the wobble uridine (U34) in tRNA; however, its specific biological roles remain unclear. In this study, we investigated the role of ALKBH8 using *Alkbh8*-knockout (*Albkh8*^−/−^) mice, which were observed to have reduced 5-methoxycarbonylmethyluridine (mcm5U) and (S)-5-methoxycarbonylhydroxymethyluridine levels; notably, the mcm5U level was partially compensated only in the brain. The results of the novel object recognition test showed reduction in time to explore a novel object in *Albkh8*^−/−^ mice; increased latency to fall in the rotarod performance test and latency to the immobility period in the forced swim test were also observed. These abnormal behaviors indicate dysfunction of the central nervous system. Furthermore, we observed reduced brain weight and ischemic pathological changes in the cerebral cortex and hippocampus in the form of weak eosin staining in the fiber tracts adjacent to the hippocampal cornu ammonis 1 region and an increase in pyramidal cells in the temporal lobe. Concordantly, we identified the differential expression of oxidative stress-related proteins and metabolites in the cerebral cortex and hippocampus using omics analyses. Finally, neurons and glial cells derived from *Albkh8*^−/−^ mice show reduced mitochondrial membrane potential. Collectively, these findings indicate that ALKBH8 maintains neural function through an oxidative stress-regulatory mechanism.

**Significance Statement**

Posttranscriptional modifications of RNA (i.e. the epitranscriptome) play an important role in several physiological functions and diseases. Neuronal regulation is also thought to be finely regulated by RNA modification proteins, as dysfunction of these proteins is known to contribute to several neurological disorders. However, the regulatory mechanism involving transfer RNA (tRNA)-modifying proteins remains poorly understood. In this study, we found that the tRNA-modifying protein AlkB homolog 8 (ALKBH8) plays an essential role in neurological functioning through oxidative stress regulation. Recently, mutations in *ALKBH8* have been detected in individuals with intellectual disability. Our findings provide novel insights into the function of tRNA-modifying enzymes in the context of the physiology of the central nervous system and the development of neurological diseases.

## Introduction

Posttranscriptional modification of messenger RNA (mRNA), transfer RNA (tRNA), and ribosomal RNA is collectively known as the epitranscriptome. The relevant chemical moieties are added or removed by proteins called “writers” or “erasers,” respectively, whereas the modified RNAs are recognized by “readers” (1, 2). Among the epitranscriptome-associated molecules, members of the AlkB homolog (ALKBH) family possess a domain similar to the 2-oxoglutarate and Fe(II)-dependent oxygenase domain (2-OG domain) of *Escherichia coli* AlkB and are thought to function as erasers. The human ALKBH family consists of nine proteins (ALKBH1–8 and fat mass and obesity associated [FTO]) that catalyze the demethylation of various RNA (3–6) as well as DNA (7, 8) and proteins (9, 10).

A growing body of evidence indicates that the epitranscriptome plays an important role in development (11), obesity (12, 13), and certain types of cancer (14). However, there have been very few studies on the functions of the ALKBH family in the central nervous system and any possible associations with neurological disorders. ALKBH1 is reported to contribute to axon regeneration (15) and hippocampus-dependent learning (16). Alkbh5 deficiency in mice results in profound and deleterious effects on cerebellar development under hypoxic conditions (17). Moreover, FTO-deficient mice show impaired learning and memory due to decreased adult neural stem cell proliferation and neuronal differentiation, as well as reduced brain size and body weight (18).

The functions of ALKBH proteins are mediated by demethylating the N^6^-methyladenosine of genes in brain cells. However, ALKBH8 has a unique molecular structure in that it contains not only the 2-OG domain commonly found in the ALKBH family, but also a methyltransferase (MT) domain that is not present in other ALKBH family proteins (19). The wobble uridine of tRNA (U34) is known to be the substrate of ALKBH8. This uridine at the first position of the anticodon region can have multiple modifications and form wobble base pairs with the corresponding mRNA codon in the ribosome to ensure precise decoding of the genetic code (20, 21). ALKBH8 methylates 5-carboxymethyluridine (cm5U) as a substrate via the MT domain and then hydroxylates it via the 2-OG domain to generate 5-methoxycarbonylmethyluridine (mcm5U) and (S)-5-methoxycarbonylhydroxymethyluridine ((S)-mchm5U) (5). These modifications at U34 are found in selenocysteine tRNA^Sec^, which is essential for the biosynthesis of selenoproteins (22, 23), including glutathione peroxidases (Gpx1, Gpx3, and Gpx6) and thioredoxin reductase 1 (24). At the cellular level, *Alkbh8* depletion caused a reduction in the mcm5U level and the accumulation of cm5U to diminish cellular survival upon exposure to DNA-damaging agents (25). Studies using *Alkbh8*-deficient mouse embryonic fibroblasts (*Alkbh8*^def^ MEFs) have shown that the induction of cellular senescence is accompanied by increased mitochondrial mass and oxygen consumption (26). Moreover, *Alkbh8*^def^ MEFs were sensitive to reactive oxygen species damage caused by H_2_O_2_ or rotenone (24). Regardless of these in vitro findings, it has been reported that *Alkbh8*-deficient mice are viable under normal conditions (27) but are significantly affected when exposed to external environmental stresses that induce lung dysfunction (28). Therefore, the physiological functions of ALKBH8 in vivo remain to be elucidated.

In this study, we investigated the biological functions of ALKBH8 using *Alkbh8*-knockout (*Albkh8*^−/−^) mice. We observed that *Albkh8*^−/−^ mice exhibited altered behavior, indicating central nervous system dysfunction. We also observed abnormal brain pathology in *Albkh8*^−/−^ mice, which may have been caused by oxidative stress. Furthermore, oxidative stress-related proteins were found to be differentially regulated in the brain of these mice. The contribution of ALKBH8 to oxidative stress regulation was also supported by metabolic changes in the methionine and glutathione pathways. Finally, we observed a reduction in mitochondrial membrane potential in primary neurons and glial cells derived from *Albkh8*^−/−^ mice.

## Results

### Generation of *Albkh8^−/−^* mouse

**Fig. 1.**
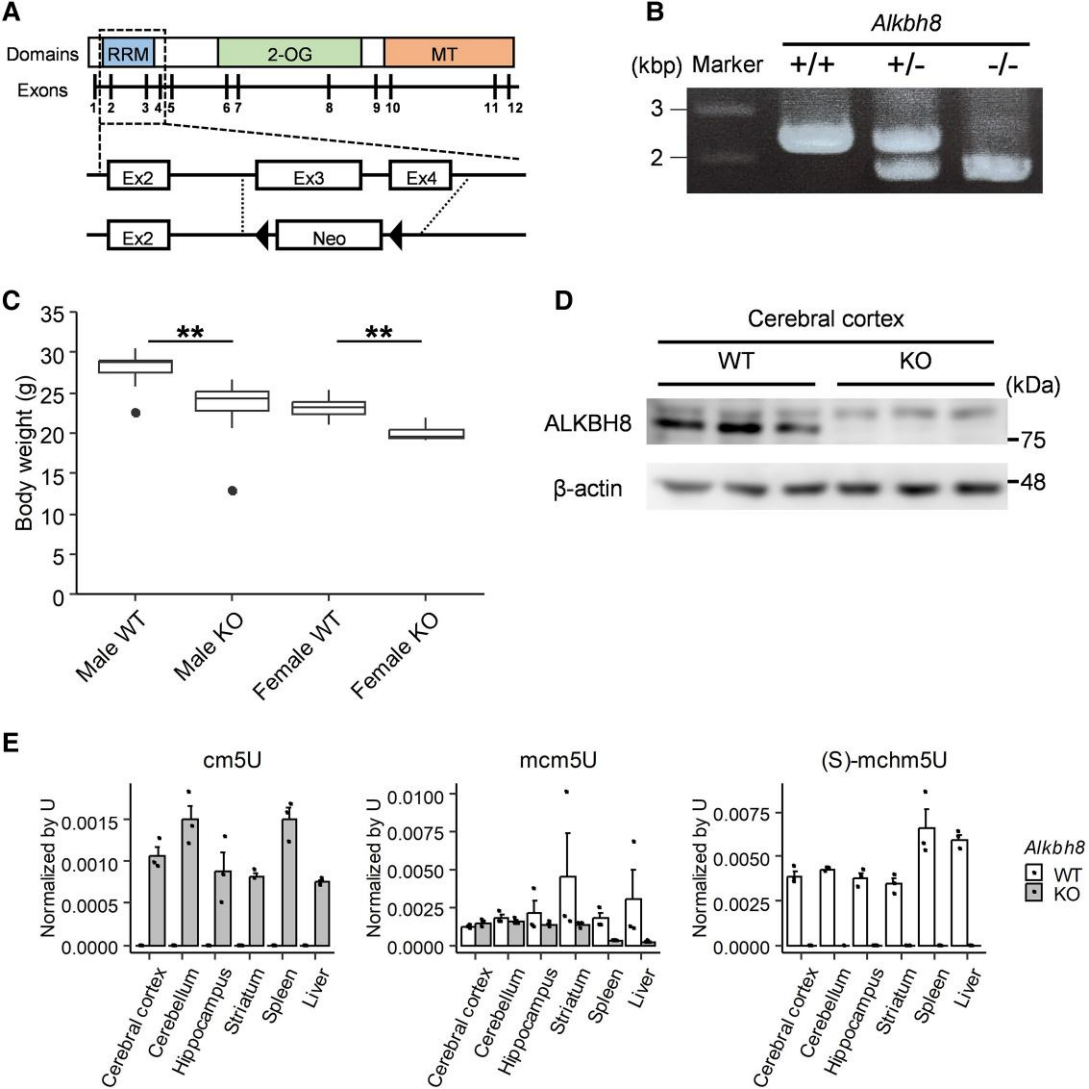
Establishment of *Alkbh8*^−/−^ mice. A) Gene-targeting strategy to delete exon 3 (Ex3) and exon 4 (Ex4) for generating *Alkbh8*^−/−^ mice. B) Heterozygous and homozygous deletions were detected using PCR targeting the Ex3 and Ex4 regions. Upper and lower bands indicate wild-type and deleted sequences, respectively. C) The body weight of *Alkbh8*^+/+^ and *Alkbh8*^−/−^ mice was measured at 15 weeks of age (*n* = 6–16, **P < 0.01, Student's t test). D) Western blotting analysis of the cerebral cortex of *Alkbh8*^+/+^ and *Alkbh8*^−/−^ mice. Tissues were lysed using N-PER reagent and probed using anti-ALKBH8 and anti-β-actin antibodies. Representative western blot images from three independent experiments are shown. E) The low-molecular-weight RNA fraction containing tRNA was extracted from each tissue indicated and degraded into nucleosides. cm5U, mcm5U, and (S)-mchm5U levels were analyzed using UHPLC-UniSpray-MS/MS (*n* = 3, mean ± SEM).

*Albkh8* ^−/−^ mice were generated by deleting exons 3 and 4, which contain the RNA recognition motif (RRM). The deletion created a frameshift to introduce a termination codon before the exons encoding the RRM domain (Fig. 1A). As a result, amino acid sequences from 89 to 709 have been removed, leaving only the segment coding for amino acids 1 to 88. Heterozygous and homozygous deletion of the gene was confirmed using PCR targeting the deleted exons 3 and 4 region (Fig. 1B). The body weights of both male and female *Albkh8*^−/−^ mice were lower than those of wild-type (*Alkbh8*^+/+^) mice (Fig. 1C). The expression of ALKBH8 in *Alkbh8*^+/+^ and *Albkh8*^−/−^ mice was assessed using western blotting (Fig. 1D). To further confirm the deficient enzymatic activity in *Albkh8*^−/−^ mice, nucleoside modifications in the low-molecular-weight RNA fraction, which contains tRNA, were quantified using mass spectrometry (MS). A significant accumulation of cm5U and a decrease in its methylated form, mcm5U, were observed in organ-derived RNA of *Albkh8*^−/−^ mice compared with that of *Alkbh8*^+/+^ mice. Furthermore, (S)-mchm5U levels were below the detection limit in *Albkh8*^−/−^ mice RNA (Fig. 1E). qPCR analysis confirmed that *Alkbh8* mRNA was expressed in all examined organs, including the brain, spleen, and liver (Fig. S1A). Interestingly, higher mcm5U levels were detected in the brain than in the spleen and liver of *Albkh8*^−/−^ mice. These results suggest that the cm5U to mcm5U conversion RNA modification is important for brain function and that a partial compensatory pathway may have operated to maintain a functional level (Fig. S1B). Figure S1C illustrates the uridine modification pathway based on information from the Modomics database (29). In addition to (S)-mchm5U, decreased 5-methoxycarbonylmethyl-2′-O-methyluridine (mcm5Um) and 5-methoxycarbonylmethyl-2-thiouridine levels were observed in the brain of *Albkh8*^−/−^ mice, but no decrease in the level of 5-carboxyhydroxymethyluridine (chm5U), which is synthesized downstream of cm5U, was observed. With regard to uridine modifications involving carbamoyl groups, the levels of 5-carbamoylmethyluridine and 5-carbamoylmethyl-2′-O-methyluridine were unchanged, while that of 5-carbamoylhydroxymethyluridine (nchm5U), which was not detectable in *Alkbh8*^+/+^ mice, was markedly increased in RNA from the brain of *Albkh8*^−/−^ mice. Although the regulatory mechanism of the uridine carbamoyl modification pathway remains unclear, the increase in nchm5U level may indicate a relative up-regulation of this pathway owing to the suppression of modifications after cm5U due to the loss of ALKBH8.

### Altered behavior of *Alkbh8^−/−^* mice

**Fig. 2.**
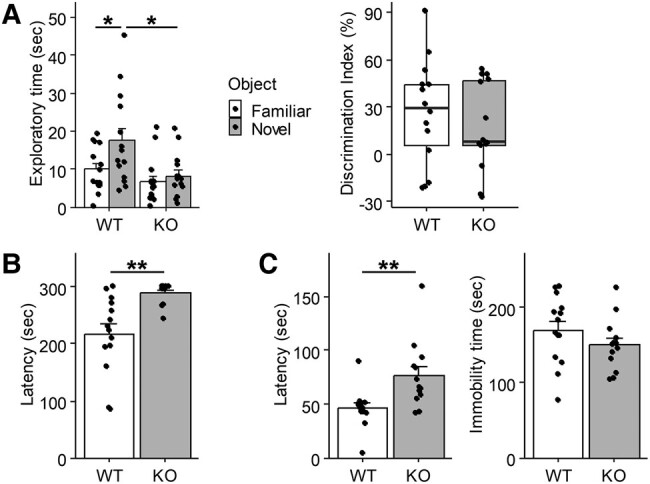
Behavioral tests on *Alkbh8*^+/+^ and *Alkbh8*^−/−^ male mice. A) NOR test. After a training session with two objects, one of them was replaced with a novel object and then the mice (male, 14–17 weeks of age) were allowed to re-explore the same field. Exploratory time for the novel object (Tn) and familiar object (Tf) were measured. The DI was calculated using the following equation: DI = (Tn − Tf)/(Tn + Tf). *n* = 14, mean ± SEM, *P < 0.05, two-way ANOVA and the Tukey–Kramer post hoc test. B) RRT. On the first 2 days, male mice (18–23 weeks of age) underwent a training session with a constant speed (16 rpm) twice a day. In the following test session, the rotarod speed was accelerated from 0 to 35 rpm and the time to fall was measured (*n* = 14 [WT] and *n* = 13 [KO], mean ± SEM, **P < 0.01, Student's t test). C) FST. Male mice (12–16 weeks of age) were placed in a glass cylinder containing water and allowed to swim for 6 min. The latency to the first immobility period and the total duration of immobility was measured (*n* = 14 [WT] and *n* = 13 [KO], mean ± SEM, **P < 0.01, Student's t test).

We noticed certain behavioral abnormalities in the established *Albkh8*^−/−^ mice during handling, including more agile movements than in *Alkbh8*^+/+^ mice. To investigate further, we conducted behavioral tests using adult male experimental mice. The novel object recognition (NOR) test is used to measure short-term recognition memory (30). After a training session, mice were allowed to freely explore a familiar object used in the training session as well as a novel object. Alkbh8^+/+^ mice spent more time exploring the novel object because of the innate preference of mice for novelty. In contrast, *Albkh8*^−/−^ mice showed little difference in exploratory time between the familiar and novel objects (Fig. 2A), as the discrimination index (DI), which is used to represent recognition memory sensitivity (30), tended to be lower in *Albkh8*^−/−^ mice, although the difference was not statistically significant. The rotarod test (RRT) is used to assess motor coordination and balance in rodents, which can be affected by drug exposure or neurological disorders (31). After the training session, the latency to fall with increasing speed was evaluated. The latency to fall of *Albkh8*^−/−^ mice was significantly prolonged compared with that of *Alkbh8*^+/+^ mice (Fig. 2B). We also conducted the forced swim test (FST), in which immobility time is often used to examine the efficacy of antidepressants; the latency to the first immobility period is also used as a parameter to differentiate between antidepressants and psychostimulants (32, 33). We observed an increase in latency to the first immobility period, but not in immobility time, in *Albkh8*^−/−^ mice (Fig. 2C). Finally, *Alkbh1^−^*^/−^ mice were reported to have decreased contextual memory in the contextual and cued fear-conditioning test (16), but we did not observe any differences in either contextual memory or cued memory in the *Albkh8*^−/−^ mice (Fig. S2). These results strongly suggest a link between the loss of ALKBH8 enzymatic activity and brain dysfunction in mice.

### Abnormal brain pathology of *Alkbh8*^−/−^ mice

**Fig. 3.**
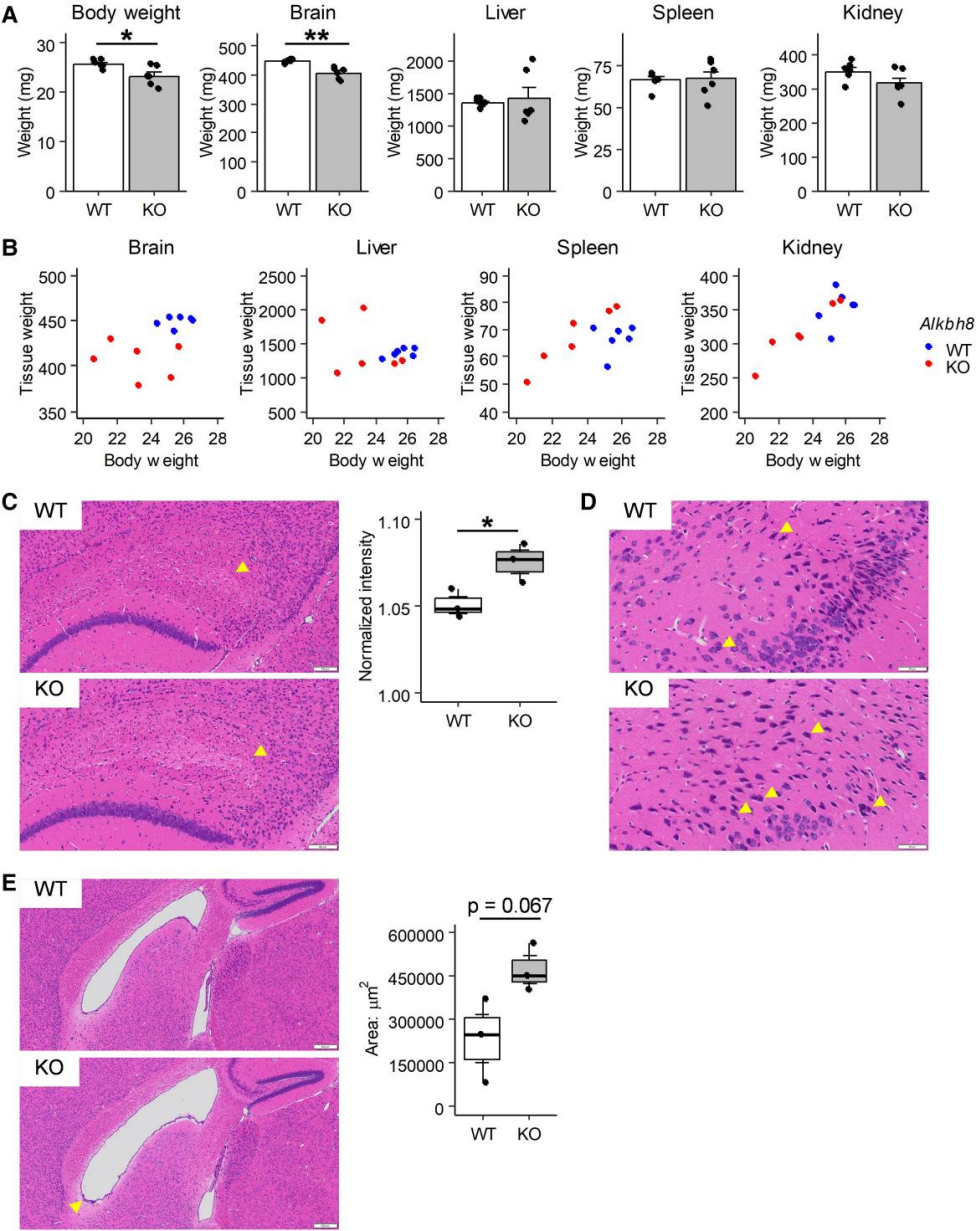
Pathological analysis of male *Alkbh8^−/−^* mice. A and B) The indicated tissues of male *Alkbh8*^+/+^ and *Alkbh8*^−/−^ mice (*n* = 6, 12 weeks of age) were excised after perfusion fixation and weighed (mean ± SEM, *P < 0.05, **P < 0.01, Student's t test). C) H&E staining was performed in sagittal sections from the excised brain. The arrowhead in the hippocampus indicates weaker eosin staining in the fiber tracts adjacent to the CA1 region in an *Alkbh8*^−/−^ mouse compared with that in an *Alkbh8*^+/+^ mouse. The intensity of eosin staining was measured by ImageJ Fiji (higher intensity shows weaker staining, *n* = 3, *P < 0.05, Student's t test). D) The arrowhead in the temporal lobe indicates the increase in shrunken and darkly stained pyknotic neurons in an *Alkbh8*^−/−^ mouse compared with those in an *Alkbh8*^+/+^ mouse. E) The arrowhead indicates enlargement of the lateral ventricle (sagittal section) and residual glial cells under the ependymal cell layer in the *Alkbh8*^−/−^ mouse. The area of the lateral ventricle was measured by ImageJ Fiji (*n* = 3, Student's t test).

Due to the differences in nucleoside modifications in the brain and altered behavior that may have been due to neurological dysfunction, we performed a histopathological examination of the brains of male *Alkbh8*^−/−^ mice. First, we confirmed that ALKBH8 was expressed in the cerebral cortex, hippocampus, and cerebellum in *Alkbh8*^+/+^ mice, and that its expression was reduced in *Alkbh8*^−/−^ mice (Figs. 1D and S3A). The whole-brain weight of *Alkbh8*^−/−^ mice was significantly lower than that of *Alkbh8*^+/+^ mice (Fig. 3A). Because the body weight of *Alkbh8^−/−^* mice is lower than *Alkbh8^+/+^* mice, we explored the correlation between body weight and organ mass. While the other tissues showed a correlation with body weight and tissue weight, the brain exhibits a consistent decrease in brain weight independent of body weight (Fig. 3B). Next, hematoxylin and eosin (H&E)-stained sagittal and coronal brain sections were evaluated. The cornu ammonis 1 (CA1) region of the hippocampus is known to be sensitive to ischemic and hypoxic stress, with shrunken and darkly stained pyknotic neurons being indicative of ischemia-induced neuronal degeneration (34). In the fiber tracts adjacent to the CA1 region of the hippocampus, statistically significant weaker eosin staining was observed in sections from *Alkbh8*^−/−^ mice than in those from *Alkbh8*^+/+^ mice (Figs. 3C and S3B). Although there were no clear differences with regard to pyramidal cells in the CA1 region, the number of pyknotic neurons at layer III or the inner layers of the temporal lobe in *Alkbh8*^−/−^ mice was much higher than those in wild-type mice (Figs. 3D and S3C). These findings indicated ischemic or hypoxic changes in the brain of *Alkbh8*^−/−^ mice.

As another pathological change, a tendency toward enlargement of the lateral ventricle and residual glial cells under the ependymal cell layer was observed in *Alkbh8*^−/−^ mice (Figs. 3E and S3D). Enlargement of the lateral ventricle indicates impaired cerebrospinal fluid (CSF) flow. The observation of a much higher number of enlarged Virchow–Robin (VR) spaces was also indicative of impaired CSF flow (Fig. S3E). The observed enlarged regions of VR space were different in each *Alkbh8*^−/−^ mouse, as summarized in Table S1.

### Changes in oxidative stress-related protein and metabolite levels in *Alkbh8*^−/−^ mice

**Fig. 4.**
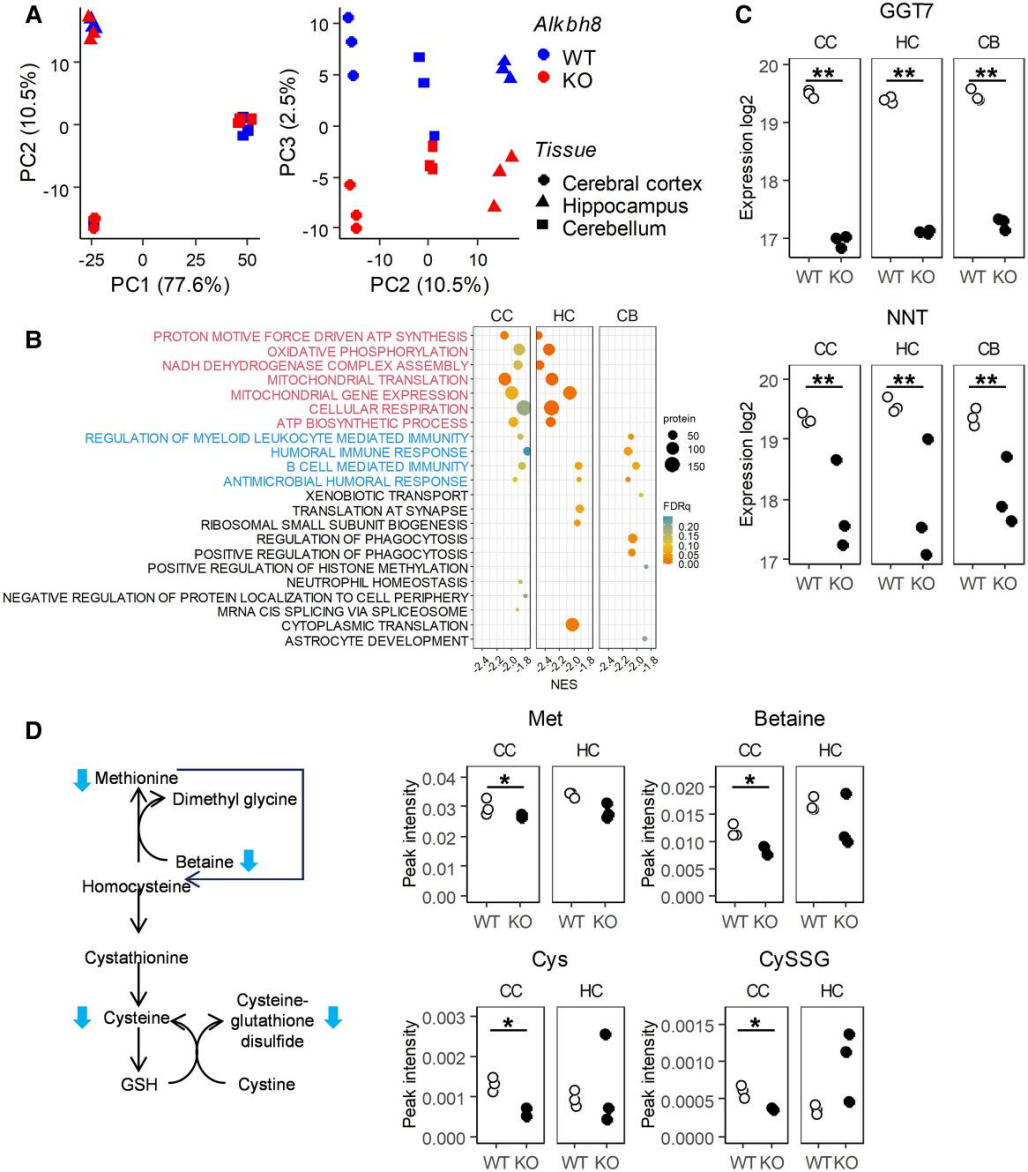
Proteomics and metabolomics analysis of *Alkbh8*^−/−^ mice. A) Proteomics analysis using mass spectrometry was conducted with peptide samples obtained from the cerebral cortex, hippocampus, and cerebellum of *Alkbh8*^+/+^ or *Alkbh8*^−/−^ mice. PCA results were plotted with components 1 (PC1) and 2 (PC2) (left panel), and components 2 (PC2) and 3 (PC3) (right panel) (*n* = 3). B) GSEA was conducted using the target GO Biological Process M5 ontology gene set, and normalized enrichment scores were plotted with the false discovery rate *q*-value. Enrichment analysis results indicate oxidative stress/mitochondria-related ontologies and immune response-related ontologies, respectively. C) Log_2_ scale expression of the GGT7 and NNT proteins in the cerebral cortex (CC), hippocampus (HC), and cerebellum (CB) of *Alkbh8*^+/+^ and *Alkbh8*^−/−^ mice (*n* = 3, **P < 0.01, Student's t test). D) Metabolomic analysis was conducted using CE-FTMS. Methionine cycle and glutathione (GSH) synthesis pathways are shown with the amount (peak MS intensity) of methionine (Met), betaine, cysteine (Cys), and cysteine–glutathione disulfide (CySSG) (*n* = 2 in the CC of KO, *n* = 3 in others, *P < 0.05, Student's t test).

ALKBH8 regulates protein translation through tRNA modification. To comprehensively examine protein expression, a proteome analysis of samples from the cerebral cortex, hippocampus, and cerebellum of adult male mice was conducted. Principal component analysis (PCA) revealed significant differences in protein expression profiles among brain tissues, which were separated by component 1 (i.e. the cerebellum from the cerebral cortex and hippocampus) and component 2 (separation of all three tissues). The difference between *Alkbh8*^+/+^ and *Alkbh8*^−/−^ mice was relatively small but distinguished by component 3 (Fig. 4A). Gene set enrichment analysis (GSEA) is a method of enrichment analysis often used to identify differentially expressed gene sets, as it allows the analysis of all genes, including those with smaller changes in expression. As the differences between *Alkbh8*^+/+^ and *Alkbh8*^−/−^ mice were small, we used GSEA in addition to fold-change analysis to identify the relevant differentially expressed proteins in this study. First, we found significant enrichment in oxidative stress- and mitochondria-related ontologies in the cerebral cortex and hippocampus of *Alkbh8*^−/−^ mice (Fig. 4B). This included the up-regulation of many mitochondrial ribosomal proteins and proteins of the mitochondrial complex I, although the changes were small (Fig. S4A). Second, ontologies related to the immune response were commonly enriched among the three tissues. Additionally, among the proteins up- or down-regulated by more than 2-fold, nicotinamide nucleotide transhydrogenase (NNT) and γ-glutamyltransferase 7 (GGT7), which are potential oxidative stress regulators, were significantly down-regulated (Figs. 4C and S4B). We also observed pathological changes in the cerebral cortex and hippocampus but not in the cerebellum (Figs. 3 and S3). This suggests that oxidative stress-related proteins enriched in the cerebral cortex and hippocampus contribute to the observed pathological changes.

We also conducted a metabolomics analysis of cerebral cortex and hippocampus tissues (Table S2). The levels of several amino acids involved in the methionine cycle and the glutathione synthesis pathway tended to be lower in the cerebral cortex of *Alkbh8*^−/−^ mice (Fig. 4D). These results, along with the proteomic changes, indicate perturbation of oxidative stress regulation, which in turn is likely to influence mitochondrial function in neurons and/or glial cells.

### Mitochondrial membrane potential in neurons and glial cells from *Alkbh8*^−/−^ mice

**Fig. 5.**
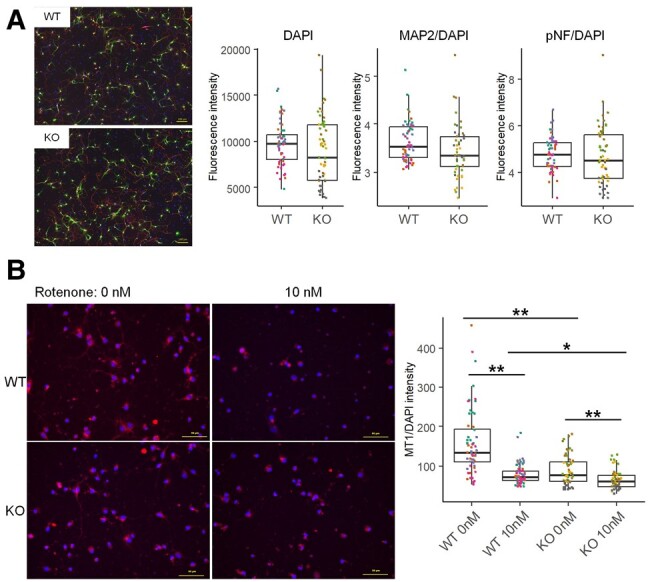
Mitochondrial function in isolated primary neurons and glial cells from *Alkbh8*^−/−^ mice. Neurons and glial cells were isolated from E17.5 embryo brains and cultured on PLL-coated plates. At 3 DIV, Ara-C was added to inhibit glial cell proliferation. Rotenone was added at 5 DIV and incubated until 7 DIV. A) Nuclei, dendrites, and axons were stained using DAPI (blue), an anti-MAP2 antibody (green), and an anti-pNF antibody (red), respectively. B) Mitochondrial membrane potential was detected using the MT-1 MitoMP reagent (red), and co-stained with DAPI (blue). The high-magnification (40×) image was used as representative image of MT-1 and DAPI, and the low-magnification (10×) image for quantification is shown in Fig. S5. The graphs on the right depict the fluorescence intensities of MAP2 and pNF (A) and MT1 (B) normalized to that of DAPI. Each plot and color indicates intensity in a microscopic field of specimens from individual mouse (15 fields in four *Alkbh8*^+/+^ or *Alkbh8*^−/−^ mice each, *P < 0.05, **P < 0.01, Bonferroni-adjusted t test).

To evaluate mitochondrial function, primary neurons and glial cells were isolated from the brains of *Alkbh8*^+/+^ and *Alkbh8*^−/−^ mice. The isolated cells were cultured and treated with cytarabine (Ara-C) to remove proliferative glial cells. On the seventh day of in vitro culture (7 DIV), neurite extension was evaluated by immunostaining dendrites and axons using the antimicrotubule-associated protein 2 (MAP2) and antiphosphoneurofilament (pNF) antibodies, respectively. We observed neurons stained with both MAP2 and pNF, as well as MAP2- and pNF-negative cells that were probably remaining glial cells. There were no significant differences in the dendrite or neurite lengths of neurons between *Alkbh8*^+/+^ and *Alkbh8*^−/−^ mice (Fig. 5A). Next, mitochondrial membrane potential was measured using MT-1 staining. The neurons and glial cells were indistinguishable from each other in this staining, but we confirmed the influence of *Alkbh8* deficiency and rotenone on the MT-1 intensity in both neuron-like cells and other cells with high-magnification image (Fig. 5B, left). The quantification analysis with low-magnification image revealed significant reduction of mitochondrial membrane potential in *Alkbh8*^−/−^ mice (mean intensity: 159.5 in *Alkbh8*^+/+^ and 86.7 in *Alkbh8*^−/−^ mice; Figs. 5B, right, and S5). The mitochondrial membrane potential was further reduced when the cells were treated with rotenone, a mitochondrial complex I inhibitor, and this reduction was more significant in *Alkbh8*^−/−^ mice than in *Alkbh8*^+/+^ mice (mean intensity: 78.2 in *Alkbh8*^+/+^ mice and 63.6 in *Alkbh8*^−/−^).

## Discussion

ALKBH8 is a “writer” enzyme that modifies the wobble uridine of tRNA and regulates protein translation, including that of selenoproteins. It has been reported that ALKBH8 has protective effects against several external stimuli, such as hydrogen peroxide, a mitochondrial complex I inhibitor, DNA-damaging agents, a glycolytic inhibitor, and environmental pollutants (24–26, 28), but specific details regarding its in vivo biological functions are limited. To investigate the in vivo biological functions of ALKBH8, we established *Alkbh8*^−/−^ mice and observed that the organs of *Alkbh8*^−/−^ mice had a marked accumulation of cm5U, whereas (s)-mchm5U level was below the detection limit. cm5U is used as a substrate by ALKBH8 and converted to mcm5U and mcm5Um by its MT activity. In *Albkh8*^−/−^ mice, the reduction in mcm5U and mcm5Um levels was significantly lower in brain tissues than in the liver and spleen. It has been reported that mcm5U is required for the translation of housekeeping selenoproteins, whereas mcm5Um is required for stress response-related selenoproteins (35). The methylation of cm5U to mcm5U and mcm5Um in the brains of *Alkbh8*^−/−^ mice may have been somewhat compensated for by some kind of mechanism to maintain the minimum essential RNA modification required for survival. Our proteomics analysis did detect several selenoproteins, but no significant differences were noted between *Alkbh8*^+/+^ and *Albkh8*^−/−^ mice in this regard. This finding is consistent with the previous report in which selenoproteins’ expression in liver, heart, kidney, lung, spleen, and brain in their *Alkbh8*^−/−^ mice was examined, and only the reduction of GPX1 in liver was observed (27). These findings indicate tissue-specific regulation of selenoprotein expression by mcm5U or mcm5Um. Although no enzyme other than ALKBH8 is known to catalyze the methylation of cm5U to mcm5U and mcm5Um, we detected a slight but statistically significant elevation in the levels of elongator acetyltransferase complex subunits (ELP1, 2, and 3) in the cerebral cortex, hippocampus, and cerebellum of *Albkh8*^−/−^ mice (Fig. S4C). ELP1, 2, and 3 assemble into a complex, bind RNA, and modify uridine into cm5U (36). These results suggest that there are some feedback mechanisms that might function to detect and compensate for the reduction of mcm5U, mcm5Um, and their downstream metabolites.

Abnormal behaviors, such as more agile movements of *Albkh8*^−/−^ mice, were observed by chance during daily rearing and led us to conduct behavioral tests. We observed a reduction in exploring time of a novel object in the NOR, increased latency to fall in the RRT, and increased latency to the first immobility period in the FST in *Albkh8*^−/−^ mice compared with those in *Alkbh8*^+/+^ mice. The NOR, RRT, and FST results are interpreted as indices of short-term recognition memory, motor coordination, and response to antidepressants, respectively. Although further detailed examination of behavioral abnormalities is required, we believe that these test results are indicative of central nervous system dysfunction in *Albkh8*^−/−^ mice. Associations between several RNA-modifying enzymes and neurological diseases have been reported. Loss-of-function mutations in NOP2/Sun RNA MT2 and MT6 (NSUN2 and NSUN6, respectively) have been identified as the cause of autosomal-recessive intellectual disability (ID) in humans (37, 38). Moreover, ELP2 mutations have been detected in patients with ID and autism spectrum disorder (39, 40). Behavioral abnormalities indicating loss of learning ability have been observed In *Alkbh1*^−/−^ mice (16). However, the tests used to detect these abnormalities differed from those used for *Albkh8*^−/−^ mice in this study (i.e. contextual and cued fear-conditioning test and Morris water maze test for *Alkbh1*^−/−^ mice and the NOR, RRT, and FST for *Albkh8*^−/−^ mice). These differences suggest that the ALKBH family and/or other components of the epitranscriptomic machinery not only work cooperatively in the central nervous system but also have specific individual functions.

To determine the cause of the observed behavioral abnormalities in *Alkbh8*^−/−^ mice, brain pathology was evaluated. *Alkbh8*^−/−^ mice showed a consistent decrease in brain weight independent of body weight compared with *Alkbh8*^+/+^ mice. It is not clear whether the decrease in brain weight was due to pathological changes. Nonetheless, some qualitative changes were also observed in the brain. The hippocampus, especially the CA1 region, is known to be vulnerable to ischemic injury (34), which causes neuronal cell death that manifests as shrunken and darkly stained pyknotic neurons. In *Alkbh8*^−/−^ mice, weak eosin staining was observed in the fiber tracts adjacent to the CA1 region of the hippocampus, and increased numbers of pyknotic neurons were observed in layer III and the inner layers of the temporal lobe but not in the CA1 region. These observations suggest that neurons in *Alkbh8*^−/−^ mice had undergone hypoxic damage, which can lead to abnormal behavior. Although this observation was relatively weak compared with the pathological changes observed in ischemia models (41), it may be consistent with the less severe abnormal behavior observed. A tendency toward enlargement of the lateral ventricles was also observed; this is a known phenotype observed in hydrocephalus and indicates a disturbance in CSF homeostasis (42). The enlarged VR spaces observed in *Alkbh8*^−/−^ mice were also thought to be a result of impaired CSF flow. These pathological phenotypes may have also resulted in the observed neuronal damage and abnormal behavior.

The findings regarding histopathological changes in *Alkbh8*^−/−^ mice associated with hypoxic damage were also supported by the analyses of changes in protein expression and metabolism. Notably, the levels of NNT and GGT7 were found to be significantly reduced. NNT is a mitochondrial protein that is involved in peroxide detoxification through nicotinamide adenine dinucleotide metabolism (43) and also has important functions in brain mitochondria (44). GGT7 is of the γ-glutamyltransferase family; these proteins are involved in glutathione metabolism, but the function of GGT7 has not been well studied although its potential activity has been predicted based on its amino acid homology with GGT1 (45). It has been reported that GGT7 expression is higher in the brain than in other tissues (46) and that it has protective effects against glioblastoma growth (47). On the other hand, GSEA identified the up-regulation of oxidative stress and mitochondria-related ontologies in *Alkbh8*^−/−^ mice, including in mitochondrial ribosomal proteins and many proteins of mitochondrial complex I. Based on our observations of reduced levels of amino acids related to glutathione synthesis and a reduction in mitochondrial membrane potential in neurons and glial cells in *Alkbh8*^−/−^ mice, the up-regulation of mitochondrial proteins in the GSEA seems to be unrelated to the up-regulation of its function. Rather, it appears that it represents a feedback signal to compensate for the reduced protective function against oxidative stress. Translational regulation via tRNA modification by ALKBH8 has not yet been fully elucidated. Several tRNAs are known to be modified by ALKBH8 (i.e. tRNA^Arg^(UCU), tRNA^Gln^(UUG), tRNA^Glu^(UUC), tRNA^Sec^(UCA), tRNA^Gly^(UCC), and tRNA^Lys^(UUU)) (48). These tRNAs are widely used for multiple protein translation and it is difficult to elucidate contribution to specific protein. Further technical advance is required to reveal direct link between these modifications and the proteins we found in this study. This study has not yet elucidated the specific oxidative stress that impacts mouse behavior. Oxidative stress can influence brain function broadly from developmental stages to adulthood (49, 50). Future research aimed at identifying the types and timing of oxidative stressors affecting behavior is expected to provide a more detailed understanding of their contribution.

In this study, we identified certain the characteristic phenotypes of *Alkbh8*^−/−^ mice, including reduced body weight and brain weight, pathological changes in the brain, and molecular changes related to oxidative stress. However, these findings are somewhat inconsistent with those of previous reports. Songe-Møller et al. (27) reported dysfunction of tRNA wobble uridine modification in *Alkbh8*^−/−^ mice, but no obvious phenotype was reported. Leonardi et al. reported that *Alkbh8*^−/−^ mice are viable and produce offspring that survive to adulthood under normal care conditions; however, they also observed an increase in oxidative stress markers under basal conditions and a higher susceptibility to lung damage when the mice were exposed to naphthalene, an environmental toxin (24, 28). In our behavioral examinations of *Alkbh8^−/−^* mice, no severe behavioral disorder or pronounced phenotypic change was observed. Had we not been careful in our observations, we may have not have noticed the differences between the wild-type and mutant mice. Nevertheless, Leonardi et al. observed significant phenotypic changes under stress induction, and Songe-Møller et al. mentioned their interest in analyses aimed at detecting minor changes in the appearance and functioning of the neural system. Although we cannot exclude the possibility of the use of a different targeting strategy against the *Alkbh8* gene (i.e. our *Alkbh8*^−/−^ mice lack the 2-OG, MT, and RRM domain, on the other hand, Songe-Møller et al. and Leonardi et al. used the deletion of 2-OG and MT domain but not RRM), there is no evidence that the RRM region alone has any function related to tRNA modification. The development of knockin mice with mutated *Alkbh8* found in human will provide new insights, particularly in terms of extrapolation to humans.

Our findings indicate that ALKBH8 plays an important role in the maintenance of central nervous function by regulating oxidative stress. Recently, multiple *ALKBH8* mutations have been identified in the causative gene of patients with ID (51–54), and the mutations were located within or near the MT domain. We generated two recombinant proteins with the *ALKBH8* mutations and confirmed that these proteins lost their MT activity, but not their 2-oxoglutarate and Fe(II)-dependent oxygenase activity. Although the detailed mechanisms by which ALKBH8 affects ID is unknown, it becomes clear from our experiments with the recombinant ALKBH8 proteins that the reported accumulation of cm5U and decrease in mcm5U, mchm5U, and mcm5Um levels in the lymphoblasts of patients are due to changes in enzyme activity caused by mutations in the MT domain of ALKBH8. However, in our *Alkbh8*^−/−^ mouse brains, we observed either unchanged or up-regulated RNA modification pathway activity, including that related to the carbamoyl group. Along with some degree of compensation of mcm5U, (S)-mchm5U, and the downstream modification pathway may be more important for the observed brain phenotype. Recently, we found that ALKBH4 contributes to the hydroxylation of mcm5U to (R)-mchm5U and its further conversion, and that the modification affects the efficacy of protein translation (55). How these modified tRNA regulate protein translation in the brain is unknown, but a comprehensive understanding of how tRNA modification and protein translation are directly affected by ALKBH8 and regulated by other related enzymes, such as ALKBH4, is required to further understand the molecular mechanisms of action of ALKBH8. Additional neurological analyses and behavioral tests are also important to link the findings of animal models to human disorders.

In conclusion, we demonstrated that ALKBH8 plays an essential role in neurological functions through an oxidative stress-regulation mechanism. Our findings also highlight the importance of several proteins and metabolites affected by ALKBH8 and (S)-mchm5U and their downstream tRNA modifications. Further elucidation of the functional mechanism of ALKBH8 is expected to contribute to the understanding of the physiological functions of the central nervous system and the pathophysiology of neurological disorders, such as ID.

## Materials and methods

### Generation of *Alkbh8*-deficient (*Alkbh8*^−/−^) mouse

The animal experiments were approved by the Animal Experimentation Committee of the Graduate School of Pharmaceutical Sciences, Osaka University. All animal experiments were performed in accordance with the relevant guidelines and regulations. The *Alkbh8* targeting vector was constructed by cloning the regions upstream of exon 3 (2,021 bp) and downstream of exon 4 (6,072 bp) into the NotI-XhoI and NheI-KpnI sites of a pPNT2 vector, respectively, resulting in neo-cassette insertion and skipping of exons 3 and 4. *Alkbh8*-knockout mice were generated using a standard gene-targeting method at the Genome Information Research Center, Osaka University. The established *Alkbh8*^−/−^ mice were backcrossed more than 12 times with C57BL/6N mice to obtain the mice then used in the experiments. The truncation of exons 3 and 4 was confirmed by PCR using forward (5′-TCC CAC TCT ATA AAG TGG GG −3′) and reverse primers (5′− TGC TTC CAG TGA TCT TGA GG −3′).

### Western blot analysis

The cerebral cortex, hippocampus, and cerebellum were dissected from *Alkbh8*^+/+^ and *Alkbh8*^−/−^ mice, and then frozen at −80 °C. The frozen samples were homogenized in N-PER reagent (Thermo Fisher Scientific) using a BioMasher II (Nippi). After centrifugation 203,805,000×*g* at 4 °C for 15 min), the supernatants were subjected to the following procedure: tissue lysates were resolved on a 7.5% sodium dodecyl sulfate–polyacrylamide gel, and proteins were then transferred to a polyvinylidene fluoride membrane (Merck Millipore). The membranes were blocked with 5% skim milk (Morinaga Milk Industry) and incubated overnight with an anti-ALKBH8 antibody (HPA038725, Sigma-Aldrich) or anti-β-actin antibody (010-27841, FUJIFILM Wako Pure Chemical). After incubation with horseradish peroxidase (HRP)-conjugated antirabbit IgG or antimouse IgG (Santa Cruz Biotechnology), the HRP signals were visualized using Amersham ECL Prime (Cytiva), and images were captured using Amersham Imager 680 (GE Healthcare).

### RNA isolation

Dissected tissues were immediately immersed in RNAprotect Tissue Reagent (QIAGEN, Hilden, Germany), and the samples were homogenized in QIAzol reagent (QIAGEN) using zirconium beads. Large and small RNA fractions were isolated using the miRNeasy Mini Kit and RNeasy MinElute Cleanup Kit (QIAGEN), respectively, according to the manufacturer's protocols. The quality of the isolated RNA was assessed using Experion RNA StdSens kit (Bio-Rad).

### Mass spectrometry analysis of small RNA modification

Small RNA modification analysis was conducted as previously described (56). Briefly, five microliters 0.1 M CH_3_COONH_4_ (pH 5.3) and 0.1 units of nuclease P1 (FUJIFILM Wako Pure Chemical) were added to 200 ng purified RNA and 1/100 volume of dG15N5 (internal standard) in 35 μL H_2_O and incubated for 2 h at 45 °C. Subsequently, 0.01 units of bacterial alkaline phosphatase (Takara Bio) were added and incubated for 2 h at 37 °C. Then, 60 μL H_2_O and 20 μL chloroform (FUJIFILM Wako Pure Chemical) were added to the mixture. The sample was vortexed, and the resulting suspension was centrifuged for 5 min at 5,000*×g*. The aqueous layer was collected and dried using a centrifugal concentrator (CC-105; TOMY). The resulting nucleoside residues were then dissolved in ultrapure water. UHPLC-UniSpray-MS/MS analyses were conducted using a Waters ACQUITY UPLC system (Waters Corp.) coupled with a Xevo TQ-XS triple quadrupole mass spectrometer (Waters Corp.).

### Forced swim test

Mice were individually placed in a glass cylinder (height: 24.0 cm, diameter: 18.5 cm) which contained water (25 ± 1 °C) at a depth of 13.0 cm. The mice were allowed to swim for 6 min, and their behavior was recorded using a video camera. After 6 min, the mice were removed, dried with a towel, and returned to their cage. The tank water was changed after each swimming session. Duration of immobility was defined as the absence of active, escape-oriented behaviors such as swimming, jumping, rearing, sniffing, or diving. Latency to the first immobility period and the total duration of immobility were measured during the 6 min test session.

### NOR test

Each mouse was habituated to an open box (30 cm × 30 cm × 35 cm) with sawdust-covered floor placed in a sound-attenuated room under dim light conditions (20 lx) for 10 min twice daily for 3 consecutive days. In the training session on day 4, two novel objects were randomly selected from three objects that were different in shape and color but similar in size, including a golf ball, a plastic toy block, and a bottle cap. The two objects were symmetrically placed on the floor, 8 cm away from the wall, and the mouse was allowed to explore freely for 10 min. In the retention session at 24 h after the training session, the mouse was allowed to explore the same field for 5 min, during which one of the familiar objects used in the training was replaced by a novel object. The performance of the each mouse was recorded using a digital camera. Exploring an object was defined as the condition in which the head of the mouse was positioned within 1 cm of the object and the mouse was facing the object. The DI was defined as the difference in exploratory time for a novel object (Tn) and a familiar object (Tf), divided by the total exploration time (i.e. DI = (Tn − Tf)/(Tn + Tf)).

### Rotarod test

The RRT was conducted using a rotarod apparatus (UGO Basile Accelerating Rotarod). The mice were placed on a rotating drum (diameter, 2 cm), and the time during which each mouse was able to maintain its balance on the drum was measured as the latency time to fall. On the first 2 days, each mouse was trained on the drum rotating at a constant speed (16 rpm) for 10 min twice a day. After this training session, each mouse was subjected to a test session. The speed of the rotarod was accelerated from 0 to 35 rpm over a 5-min period.

### Histological analysis of the brain

Mice were anesthetized by isoflurane inhalation, transcardially perfused with phosphate-buffered saline (PBS) and 4% paraformaldehyde (PFA) dissolved in PBS. The whole brains were excised and postfixed in 4% PFA in PBS for 24 h. After fixation, 4% PFA was replaced with 70% ethanol until histological analysis. H&E staining was performed by Applied Medical Research Laboratory, Osaka, Japan.

The quantification was conducted with ImageJ Fiji (ver. 1.54c) (57). The intensity of eosin staining in the fiber tracts adjacent to the CA1 region was quantified by normalizing it using the intensity of the adjacent regions where differences are imperceptible. The size lateral ventricle was quantified by automatically detecting its area with ImageJ.

### Proteomics analysis

Protein lysates were prepared using the same method used that used for western blotting analysis. Protein digestion was conducted using the EasyPrep Mini MS Sample Prep Kit (Thermo Fisher Scientific), and the peptides were resolved in 0.1% formic acid.

Online liquid chromatography (LC)–MS was performed using an Easy-nLC 1200 system coupled with an Orbitrap Eclipse Tribrid mass spectrometer (Thermo Fisher Scientific). Samples were trapped on a C18 guard-desalting column (Thermo Fisher Scientific, Acclaim PepMap 100, 75 μm × 2 cm, nanoViper, C18, 5 µm, 100 Å), and separated on a 12-cm long C18 column (Nikkyo Technos, C18, 3 μm, 75 μm × 12 cm). The nanocapillary solvent A consisted of 100% water and 0.1% formic acid; solvent B consisted of 20% water, 80% acetonitrile, and 0.1% formic acid. At a constant flow rate of 0.3 μL/min, the curved gradient went from 6% B up to 31% B, followed by a steep increase to 90% B in 10 min. The eluted peptide samples were analyzed using a data-independent acquisition (DIA) method. For MS1, *m*/*z* was set from 500 to 1,100, mass resolution was 24,000, AGC target was 500%, maximum injection time was 50 ms, and the data type was centroid. MS2 was acquired in quadrupole isolation mode with the isolation window set to 10 *m*/*z*, 25% HCD collision energy, 120,000 mass resolution with Orbitrap detection, 2,000% AGC target, maximum injection time, and centroid data type. Raw data files were processed in library-free mode in DIA-NN (version 1.8.1). Trypsin/P allowed a maximum of one missed cleavage, N-terminal methionine removal for variable modifications, and fixed carbamidomethylation on cysteine. Peptide lengths ranged from 7 to 30 amino acids. The precursors ranged from 300 to 1,800, and the fragment ions ranged from 200 to 1,800. Both MS1 and MS2 mass accuracies were set to automatic determination. Protein inference was set to “Protein names (from FASTA)” and the “Heuristic protein inference” option and MBR (between-run) were employed. The RT-dependent and Robust LC (high precision) options were selected for quantification. The MS data have been deposited in the jPOST repository (jPOST ID: JPST002354) (58).

Data processing was performed using the Perseus computational platform (59). Briefly, the proteins detected in all six samples were selected as target proteins for differential expression analysis (6,991 of 7,830 detected proteins). Log_2_-transformed data were quantile normalized, and PCA was conducted with the normalized data using Perseus software. Differentially expressed proteins were defined as those with fold-change ≥1.5 and *P* < 0.05. GSEA software developed by UC San Diego and Broad Institute (60, 61) was used for enrichment analysis, in which the GO Biological Process ontology M5 ontology was used as a target gene set.

### Metabolomics analysis

The cerebral cortex and hippocampus of *Alkbh8*^+/+^ and *Alkbh8*^−/−^ mice (male, 12 weeks of age, *n* = 3, respectively) were removed and frozen at −80 °C. Metabolite extraction and capillary electrophoresis-Fourier transform MS (CE-FTMS) were performed by Human Metabolome Technologies, Inc. One of the cerebral cortex data points from an *Alkbh*^8−/−^ mice, which showed an obvious abnormal distribution of expression intensity, was excluded from the differential expression analysis. Differentially expressed metabolites were defined as those with fold-change ≥1.2 and *P* < 0.05.

### Primary cultures of mouse neurons and immunocytochemistry

The cerebral cortex, including the hippocampus, was isolated from E17.5 embryos obtained from pregnant *Alkbh8*^+/+^ female mice interbred with *Alkbh8*^+/+^ male mice or *Alkbh8*^−/−^ female mice interbred with *Alkbh8*^−/−^ male mice and then dissociated using a Neural Tissue Dissociation Kit (Miltenyi Biotech), according to the manufacturer's protocol. The isolated cells were cultured in Neurobasal Medium (Thermo Fisher) supplemented with B-27 Supplement (Thermo Fisher), penicillin, and streptomycin (FUJIFILM Wako Pure Chemical) on 0.1 mg/mL poly-l-lysine (PLL)-coated culture plates. Ara-C was added at a final concentration of 5 μM at 3 DIV, and rotenone was added at a final concentration of 10 nM at 5 DIV. At 7 DIV, the cells were fixed with 4% PFA and incubated overnight with anti-MAP2 antibody (#5622, Millipore) and anti-pNF antibody (#801601, BioLegend), and then with AlexaFluor 488 antirabbit IgG (Invitrogen) and AlexaFluor 568 antimouse IgG (Invitrogen), respectively. On the same day, mitochondrial membrane potential was estimated using the MT-1 MitoMP Detection Kit (Dojindo). Fluorescent images (*n* = 4, 15 fields from each mouse) were captured and the intensity was quantified using a BZ-X800 fluorescence microscope (Keyence).

### Materials and methods in supporting information

#### qPCR analysis

Total RNA was extracted from tissue using the miRNeasy Mini Kit (QIAGEN). The total RNA panel of C57 strain brain tissues was purchased from Zyagen. cDNA was synthesized from total RNA using the PrimeScript RT Reagent Kit (Takara Bio). *Alkbh8* and *Actb* gene expression levels were detected using qPCR. Target sequences were amplified using the primers *Alkbh8* forward: 5′-TCC TGT CAG AAG TGG GTC TTG TG-3′, *Alkbh8* reverse: 5′- GAG ACG GGC AAG TTC TTG GAG-3′, *Actb* forward: 5′-ACC CAG GCA TTG CTG ACA GG-3′, and *Actb* reverse: 5′- GAG TAC TTG CGC TCA GGA GG-3′. Amplicons were detected using the THUNDERBIRD SYBR qPCR Mix reagent (TOYOBO). The *Alkbh8* expression was normalized to that of *Actb* expression in each sample.

#### Contextual and cued fear-conditioning test

The test was performed using fear-conditioning chambers (30 × 24 × 21 cm; MedAssociates, Inc., St Albans, VT, USA) with a MedAssociates Video Freeze system. On day 1, mice were habituated for 150 s, after which conditioning was conducted using five times tones (30 s, 2.8 kHz, 80 dB) followed by an electric foot shock (2 s, 0.5 mA) with a 90-s intertrial interval. Freezing was recorded during both the baseline period and each tone period. Twenty-four hours later (day 2), the mice were placed in the same conditioning chamber for the context fear test. No stimuli were presented during this period; freezing was recorded throughout the 8-min test. For the cued fear test on day 3, mice were placed in a different chamber with a dark roof-like triangular ceiling and grid floor covering. The mice were habituated to the novel environment for 150 s, and then five tones (30 s, 2.8 kHz, 80 dB) were presented with a 90-s intertrial interval. Freezing was recorded during both the baseline period and each tone period. The data are expressed as the average values for each five-tone period.

## Supplementary Material

pgae115_Supplementary_Data

## Data Availability

All study data are included in the article and SI Appendix.
