# Supplementary material for: ALKBH8 contributes to neurological function through oxidative stress regulation
Source: PNAS Nexus. 2024 Mar 28;3(3):pgae115. doi: 10.1093/pnasnexus/pgae115 (PMC10978050; doi:10.1093/pnasnexus/pgae115)
Supplement: pgae115_Supplementary_Data [file pgae115_supplementary_data.zip › PNASNEXUS-PNASNEXUS-2023-01185RR-s01.docx]

**
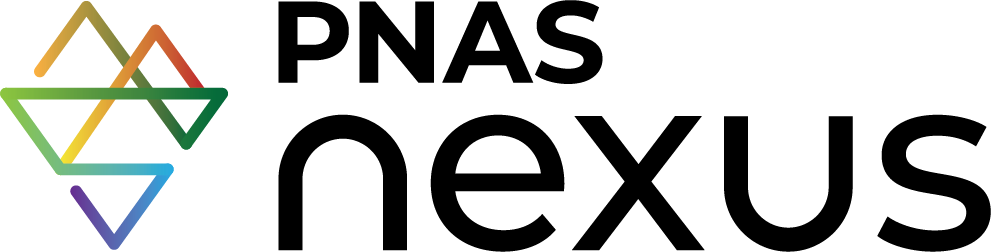
**

**Supplementary Information for**

ALKBH8 contributes to neurological function through oxidative stress regulation

Kohei Honda, Hiroaki Hase*, Sayaka Tanikawa, Katsuya Okawa, Manami Nakai, Kaori Kitae, and Kazutake Tsujikawa

*Hiroaki Hase

Laboratory of Molecular and Cellular Physiology, Graduate School of Pharmaceutical Sciences, Osaka University, 1- 6 Yamadaoka, Suita, Osaka 565-0871, Japan

Phone: +81 6-6879-8192

Email: hase-h@phs.osaka-u.ac.jp

**This PDF file includes:**

Figures S1 to S5

Tables S1

Fig. S1. *Alkbh8* expression in mouse tissues and uridine modifications in *Alkbh8*^-/-^ mice. (A) *Alkbh8* expression in some organs (left) and brain tissues (right) was assessed using qPCR (n=3, mean + SD). (B) Low MW RNA fraction containing tRNA was extracted from each tissue and degraded into nucleosides. RNA modifications were analyzed using UHPLC-UniSpray-MS/MS (n=3, mean + SEM). (C) Up- or down-regulation of uridine modifications in *Alkbh8*^-/-^ mice are summarized in the modification scheme of uridine.

Fig. S2. Contextual and cued fear conditioning test. On day 1, conditioning was conducted using five times tones followed by an electric foot shock, and fear acquisition in 14 male *Alkbh8*^+/+^ and *Alkbh8*^-/-^ mice (13–18 weeks of age) was confirmed. On day 2, the mice were placed in the same conditioning chamber to assess contextual fear. On day 3, mice were placed in a different chamber and exposed to the five tones to assess cued fear. Freezing time was measured using the MedAssociates VideoFreeze system (mean ± SEM, Student’s *t*-test).

Fig. S3. Pathological analysis of individual male Alkbh8^-/-^ mice. Images from two other mice (in addition to those in Figure 3C-E) are shown. (A) ALKBH8 deficiency was confirmed using western blot analysis of tissue lysates from the hippocampus and cerebellum of *Alkbh8*^+/+^ or *Alkbh8*^-/-^ mice (n=3). (B) The arrowhead in the hippocampus indicates weaker eosin staining in the fiber tracts adjacent to the CA region of *Alkbh8*^-/-^ mice compared to that in *Alkbh8*^+/+^ mice. (C) The arrowheads in the temporal lobe indicate an increase in shrunken and darkly stained pyknotic neurons in *Alkbh8*^-/-^ mice compared to those in *Alkbh8*^+/+^ mice. (D) The arrowhead indicates enlargement of the lateral ventricle (sagittal section) and residual glial cells under the ependymal cell layer in *Alkbh8*^-/-^ mice. (E) More enlarged Virchow-Robin (VR) spaces were observed in *Alkbh8*^-/-^ mice than in *Alkbh8*^+/+^ mice. The labels “WT-1–3” and “KO-1–3” are used for individual mice. The WT-1 mouse was used as the normal control.

Fig. S4. Proteomics analysis of the brain of *Alkbh8^-/-^* mice (A) Enrichment plot and heat map of significantly enriched proteins were derived using the GSEA software. Ontologies of “Protein motive force driven ATP synthesis” and “Mitochondrial translation” are examples of those associated with enriched proteins in the cerebral cortex and hippocampus of *Alkbh8* -/- mice compared to those in *Alkbh8*^+/+^ mice. (B) Differentially expressed proteins in the cerebral cortex, hippocampus, and cerebellum are depicted using volcano plots. Vertical and horizontal lines indicate the >=1.5 or <=1.5 fold-change thresholds and p<0.05, respectively. The names of proteins with >=2 or <= fold-change in expression are also provided. (C) log_2_ scale expression of ELP1, ELP2, and ELP3 in the cerebral cortex (CC), hippocampus (HC), and cerebellum (CB) of *Alkbh8*^+/+^ and *Alkbh8*^-/-^ mice (n=3, **p<0.01, Student’s *t*-test).

Fig. S5. Mitochondrial function in isolated primary neurons and glial cells from *Alkbh8*^-/-^ mice. Neurons and glial cells were isolated from E17.5 embryo brains and cultured on PLL-coated plates. At 3 DIV, Ara-C was added to inhibit glial cell proliferation. Rotenone was added at 5 DIV and incubated until 7 DIV. Mitochondrial membrane potential was detected using the MT-1 MitoMP reagent (red), and co-stained with DAPI (blue). Presenting representative low-magnification (10x) images for quantitative analysis.

Table S1. Summary of enlarged Virchow-Robin (VR) space

| Region | WT-1 | WT-2 | WT-3 | KO-1 | KO-2 | KO-3 |
| --- | --- | --- | --- | --- | --- | --- |
| Locus ceruleus | - | - | - | - | ++ | ++ |
| Substantia nigra | - | ++ | + | ++ | - | - |
| Cerebellum | - | - | + | - | - | - |
| Cerebral ventricle | - | - | - | ++ | - | + |
| Thalamus | - | + | + | - | - | + |
| Temporal lobe | - | - | - | + | ++ | + |

Note: Severity of VR space was evaluated in each region of H&E staining samples and assessed as follows:

-: not observed, +: mild, ++: severe
